# Supplementary material for: Phosphorylation and O-GlcNAcylation at the same α-synuclein site generate distinct fibril structures
Source: Nat Commun. 2024 Mar 27;15:2677. doi: 10.1038/s41467-024-46898-1 (PMC10973503; doi:10.1038/s41467-024-46898-1)
Supplement: Supplementary file 1 — Supplementary Information [file 41467_2024_46898_MOESM1_ESM.pdf]

Supplementary Information for

Phosphorylation and O-GlcNAcylation at the same α-synuclein site generate distinct fibril structures

Jinjian Hu[1†], Wencheng Xia[2†], Shuyi Zeng[3,4†], Yeh-Jun Lim[1†], Youqi Tao[3,4], Yunpeng Sun[2], Lang Zhao[1], Haosen Wang[1], Weidong Le[5,6], Dan Li[3,4], Shengnan Zhang[2], Cong Liu[2,7*], Yan-Mei Li[1*]

[1]Key Laboratory of Bioorganic Phosphorus Chemistry and Chemical Biology (Ministry of Education), Department of Chemistry, Tsinghua University, Beijing, 100084, China.

[2]Interdisciplinary Research Center on Biology and Chemistry, Shanghai Institute of Organic Chemistry, Chinese Academy of Sciences, Shanghai, 201210, China.

[3]Bio-X Institutes, Key Laboratory for the Genetics of Developmental and Neuropsychiatric Disorders (Ministry of Education), Shanghai Jiao Tong University, Shanghai, 200030, China.

[4]Zhangjiang Institute for Advanced Study, Shanghai Jiao Tong University, Shanghai, 200240, China.

[5]Shanghai University of Medicine & Health Sciences Affiliated Zhoupu Hospital, Shanghai, 201318, China.

[6]Center for Clinical and Translational Medicine, Shanghai University of Medicine and Health Sciences, Shanghai, 201318, China.

[7]State Key Laboratory of Chemical Biology, Shanghai Institute of Organic Chemistry, Chinese Academy of Sciences, Shanghai, 200032, China.

[†] J.H., W.X., S.Z., and Y.-J.L. contribute equally to this work.

*To whom correspondence should be addressed. Email: liym@mail.tsinghua.edu.cn or liulab@sioc.ac.cn

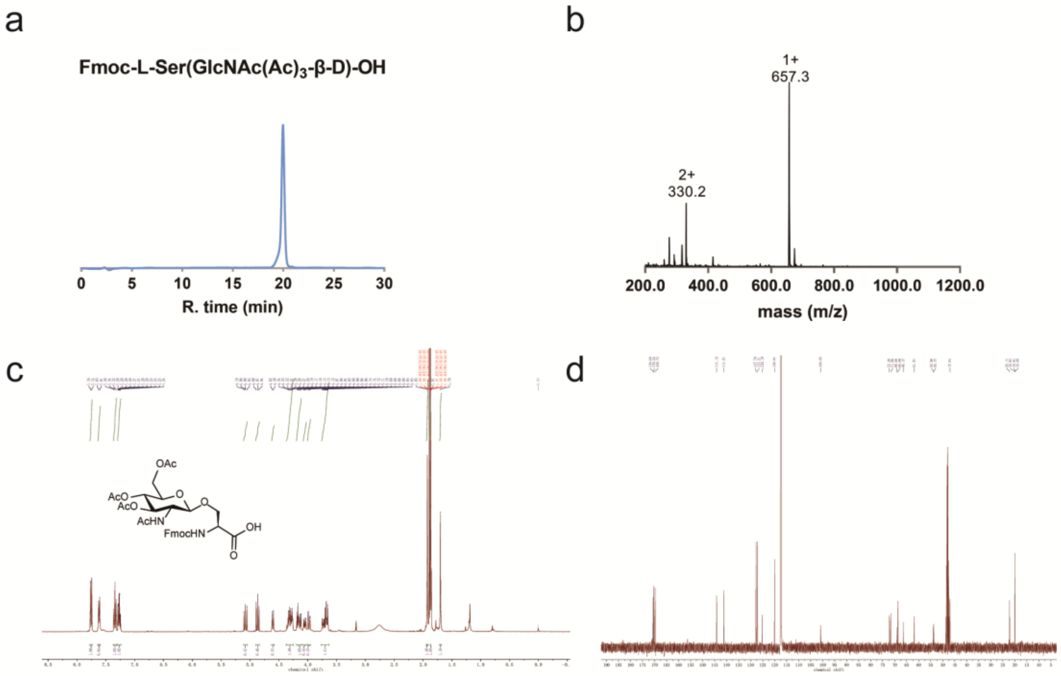

Supplementary Fig. 1. Characterization of Fmoc-L-Ser(GlcNAc(Ac)$_3$-β-D)-OH.
a Analytical HPLC result of Fmoc-L-Ser(GlcNAc(Ac)$_3$-β-D)-OH. Retention time is about 19.95 min in a linear gradient of 50-90%B for 30 min (HPLC solvent A: water, 0.06% TFA; B: 80% CH_3CN/water, 0.06% TFA). **b** ESI-MS characterization of Fmoc-L-Ser(GlcNAc(Ac)$_3$-β-D)-OH. Calculated mass: 656.6 Da, observed mass: 656.3 Da. **c** ^{1}H NMR characterization of Fmoc-L-Ser(GlcNAc(Ac)$_3$-β-D)-OH (400 MHz, Acetonitrile-*d$_3$*) δ 7.75 (d, J = 7.6 Hz, 2H), 7.62 (d, J = 7.5 Hz, 2H), 7.34 (t, J = 7.5 Hz, 2H), 7.27 (tdd, J = 7.4, 3.6, 1.2 Hz, 2H), 5.08 (dd, J = 10.6, 9.4 Hz, 1H), 4.95 – 4.80 (m, 1H), 4.60 (d, J = 8.5 Hz, 1H), 4.37 – 4.25 (m, 3H), 4.20 – 4.11 (m, 2H), 4.05 (dd, J = 10.5, 4.6 Hz, 1H), 3.98 (dd, J = 12.3, 2.5 Hz, 1H), 3.76 – 3.63 (m, 3H), 1.93 (s, 3H), 1.87 (s, 2H), 1.70 (s, 2H). **d** ^{13}C NMR characterization of Fmoc-L-Ser(GlcNAc(Ac)$_3$-β-D)-OH (400 MHz, Acetonitrile-*d$_3$*) δ 170.60, 170.32, 169.73, 144.12, 141.21, 127.79, 127.21, 125.19, 120.04, 100.83, 72.20, 71.66, 69.08, 68.69, 66.47, 61.91, 53.90, 53.77, 47.04, 22.14, 19.94, 19.91, 19.88. Source data are provided as a Source Data file.

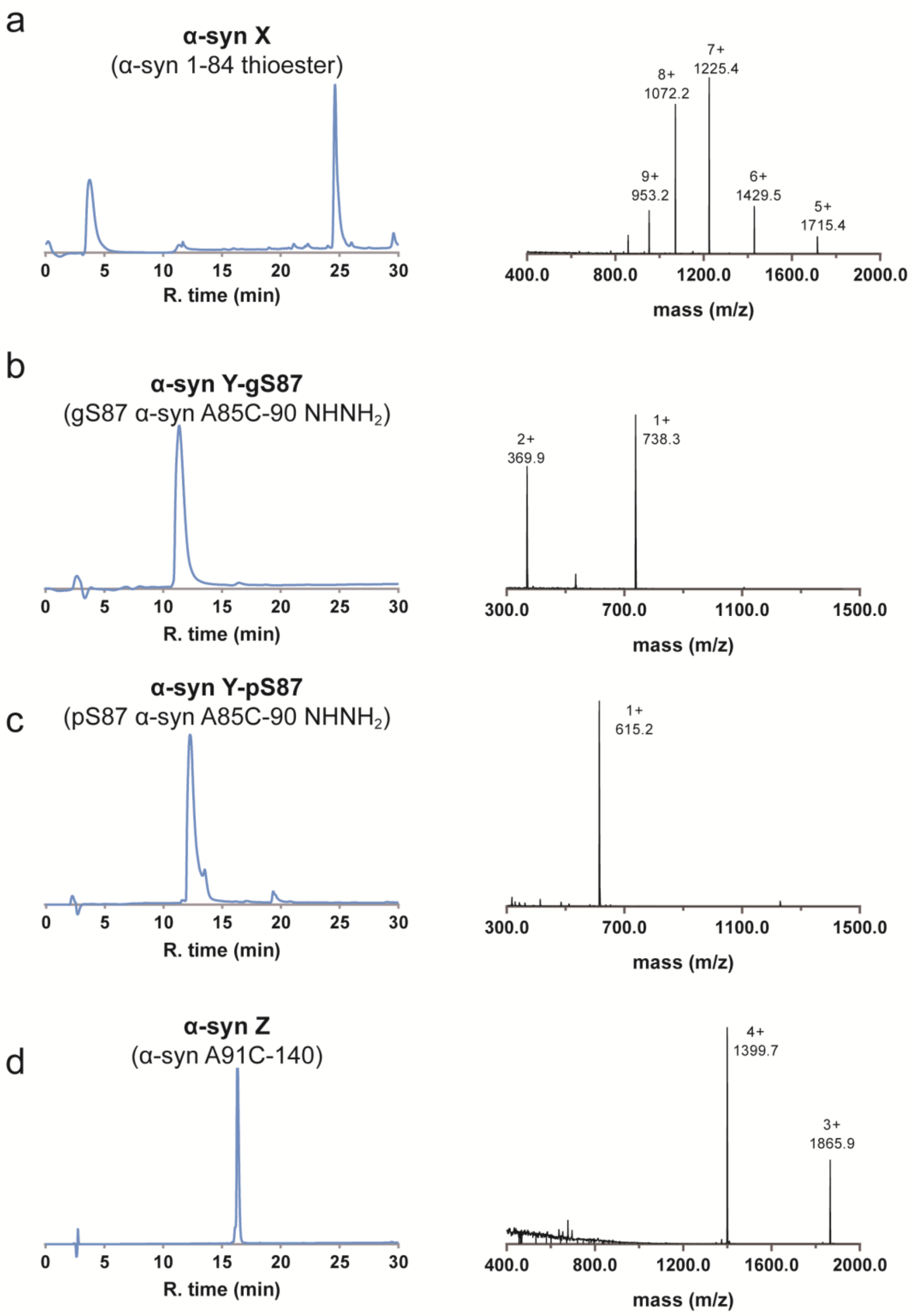

Supplementary Fig. 2. Characterization of the synthetic segments: X, Y, and Z.
a Characterization of α-syn X (MesNa thioester). Left: analytical HPLC result of α-syn X in Buffer. Retention time is about 24.62 min in a linear gradient of 30-70%B for 30 min; Right: ESI-MS characterization of α-syn X (MesNa thioester). Calculated mass: 8569.7 Da, observed mass: 8570.2 Da. **b** Characterization of α-syn Y-gS87. Left: analytical HPLC result of α-syn Y-gS87. Retention time is about 11.36 min in a linear gradient of 5-25%B for 30 min; Right: ESI-MS characterization of α-syn Y-gS87. Calculated mass: 737.3 Da observed mass: 737.5 Da. **c** Characterization of α-syn Y-

pS87. Left: analytical HPLC result of α-syn Y-gS87. Retention time is about 12.25 min in a linear gradient of 5-25%B for 30 min; Right: ESI-MS characterization of α-syn Y-pS87. Calculated mass: 614.2 Da, observed mass: 614.2 Da. **d** Characterization of α-syn Z. Left: analytical HPLC result of α-syn Z. Retention time is about 16.31 min in a linear gradient of 30-70%B for 30 min; Right: ESI-MS characterization of α-syn Z. Calculated mass: 5594.0 Da, observed mass: 5594.7 Da. Source data are provided as a Source Data file.

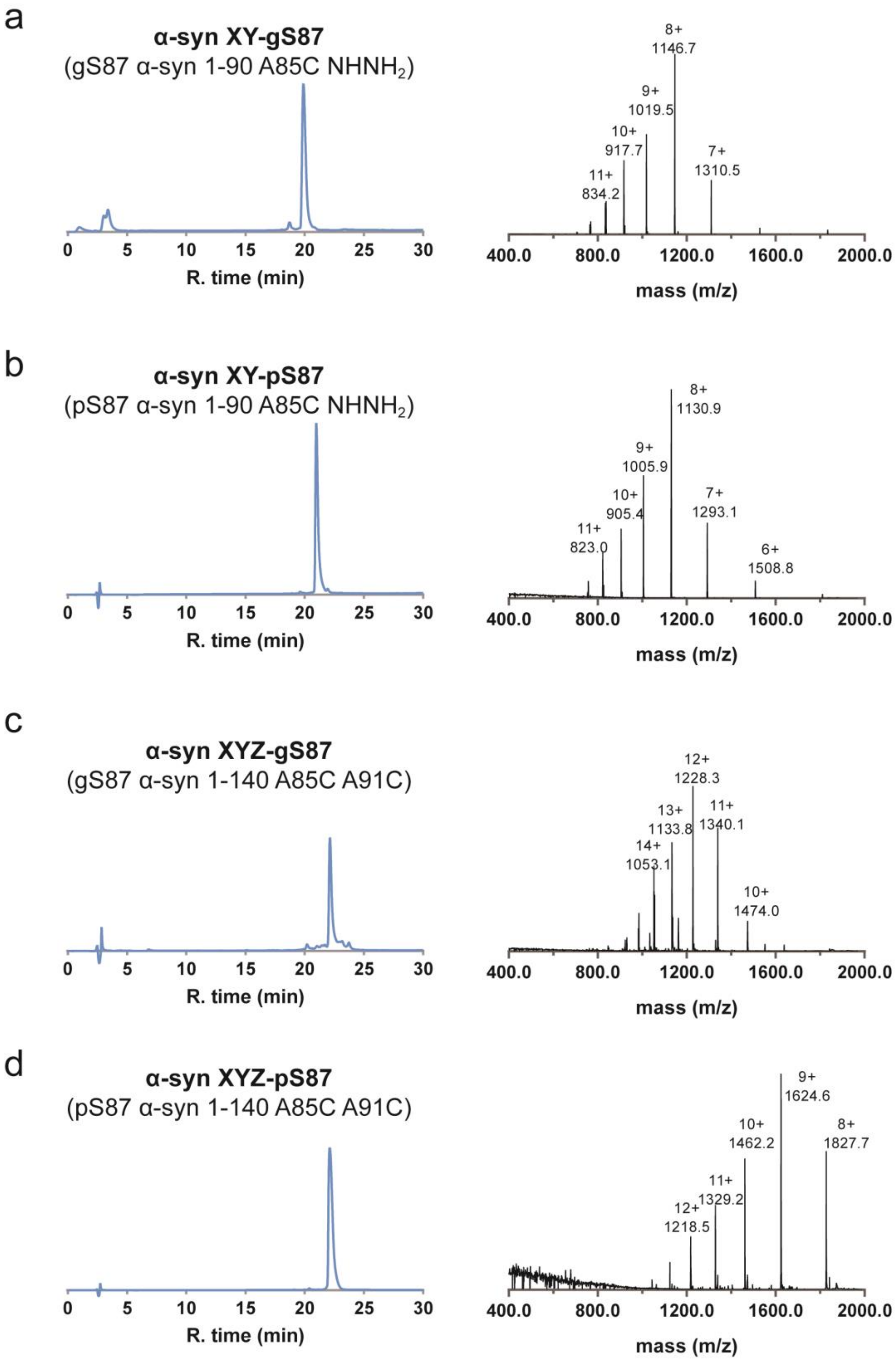

Supplementary Fig. 3. Characterization of the synthetic segments: XY and XYZ. **a** Characterization of α-syn XY-gS87. Left: analytical HPLC result of α-syn XY-gS87. Retention time is about 19.88 min in a linear gradient of 30-70%B for 30 min; Right: ESI-MS characterization of α-syn XY-gS87. Calculated mass: 9165.4 Da observed mass: 9165.8 Da. **b** Characterization of α-syn XY-pS87. Left: analytical HPLC result

of α-syn XY-pS87. Retention time is about 20.98 min in a linear gradient of 30-70%B for 30 min; Right: ESI-MS characterization of α-syn XY-pS87. Calculated mass: 9042.3 Da, observed mass: 9043.7 Da. **c** Characterization of α-syn XYZ-gS87. Left: analytical HPLC result of α-syn XYZ-gS87. Retention time is about 22.13 min in a linear gradient of 30-70%B for 30 min; Right: ESI-MS characterization of α-syn XYZ-gS87. Calculated mass: 14727.3 Da, observed mass: 14729.2 Da. **d** Characterization of α-syn XYZ-pS87. Left: analytical HPLC result of α-syn XYZ-pS87. Retention time is about 22.11 min in a linear gradient of 30-70%B for 30 min; Right: ESI-MS characterization of α-syn XYZ-pS87. Calculated mass: 14604.3 Da, observed mass: 14612.1 Da. Source data are provided as a Source Data file.

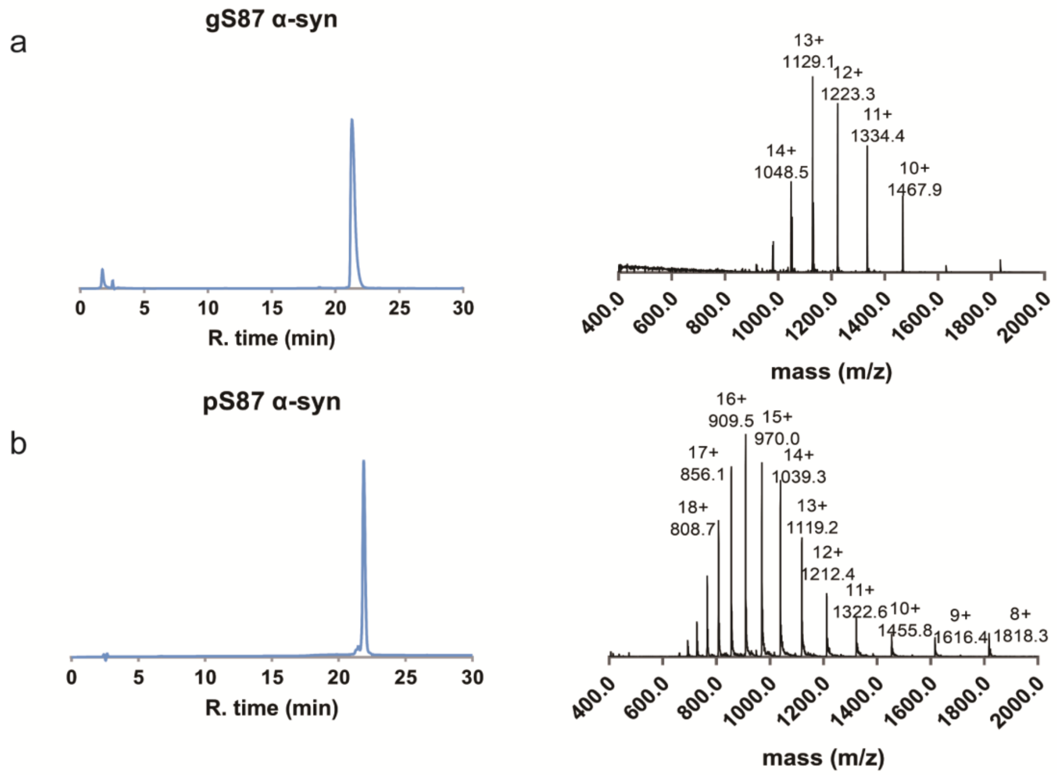

Supplementary Fig. 4. Characterization of the synthetic pS87 α-syn and gS87 α-syn.

a Characterization of gS87 α-syn. Left: analytical HPLC result of gS87 α-syn. Retention time is about 21.28 min in a linear gradient of 30-70%B for 30 min; Right: ESI-MS characterization of gS87 α-syn. Calculated mass: 14663.3 Da, observed mass: 14666.1 Da. **b** Characterization of pS87 α-syn. Left: analytical HPLC result of pS87 α-syn. Retention time is about 21.46 min in a linear gradient of 30-70%B for 30 min; Right: ESI-MS characterization of pS87 α-syn. Calculated mass: 14540.1 Da observed mass: 14536.4 Da. Source data are provided as a Source Data file.

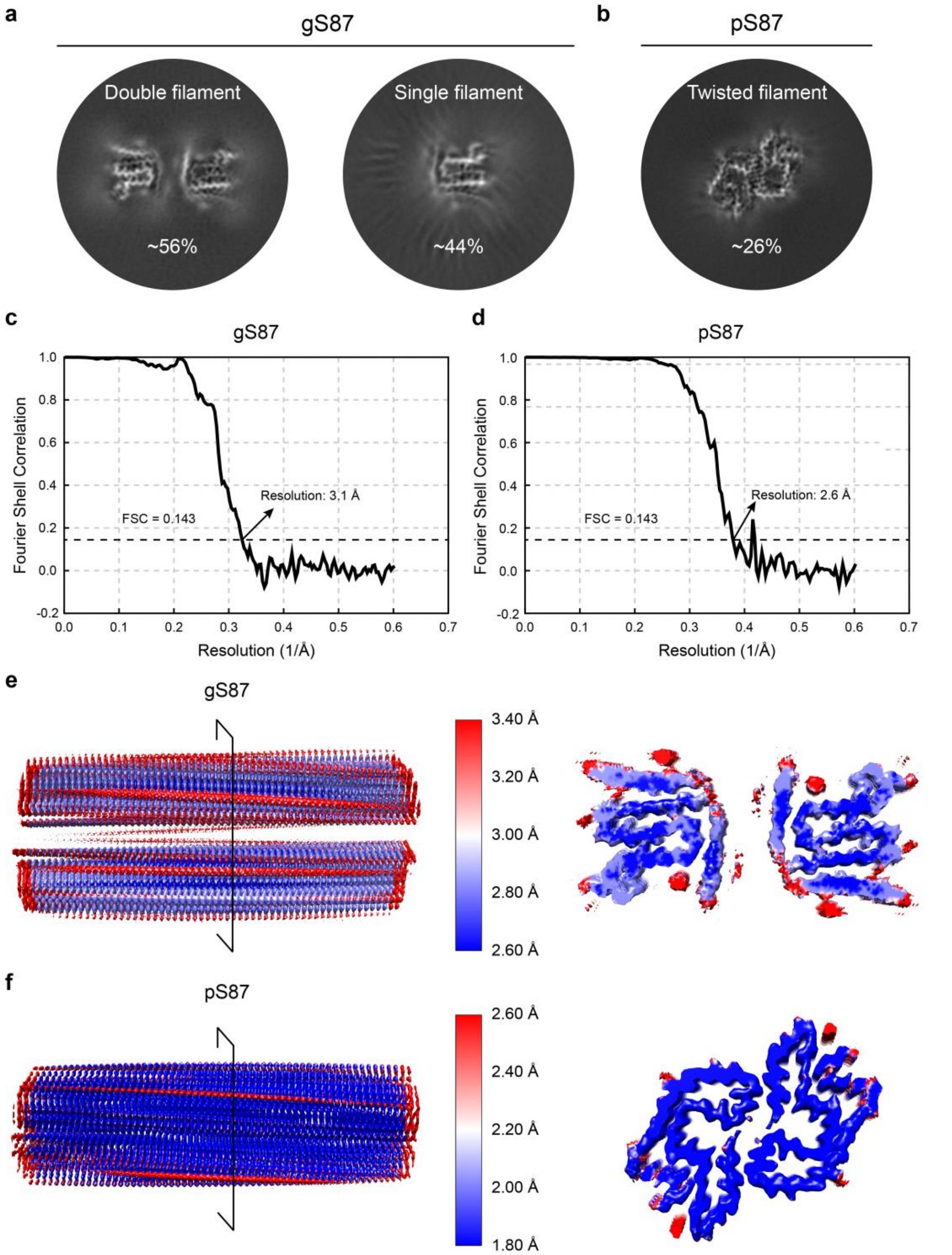

Supplementary Fig. 5. Cryo-EM structure determination of the gS87 and pS87 α-syn fibrils.

a 3D classification results of the double filament polymorph (~56%) and the single filament polymorph (~44%) of the gS87 α-syn fibril. **b** 3D classification result of the twisted filament polymorph (~26%) of the pS87 α-syn fibril. **c, d** Gold-standard Fourier shell correlation (FSC) curves of the density maps of the gS87 (**c**) and pS87 (**d**) α-syn fibrils. **e**, **f** Local resolution estimations of the density maps of gS87 (**e**) and pS87 (**f**).

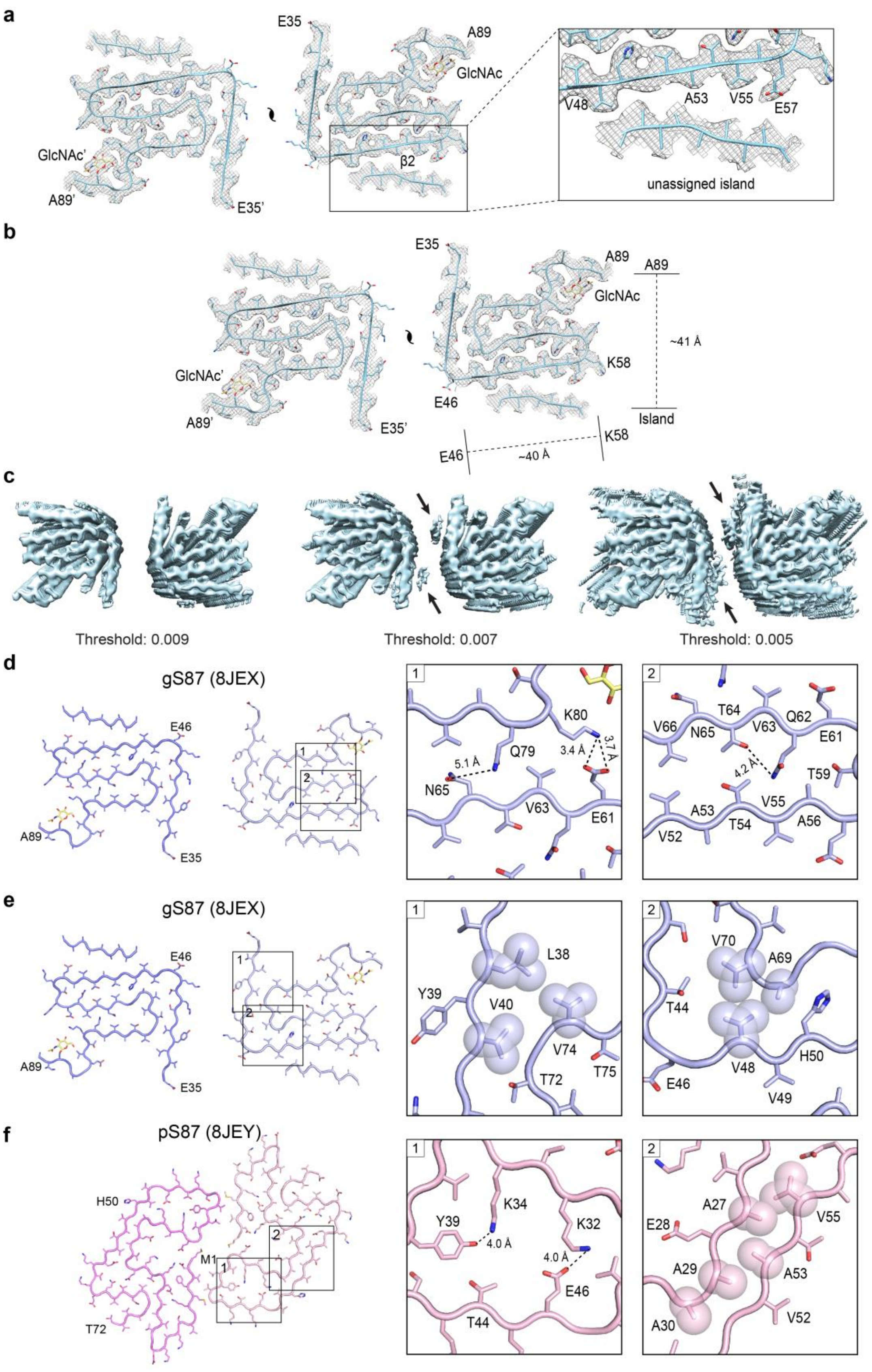

Supplementary Fig. 6. Structural analysis of the gS87 and pS87 α-syn fibrils.
a In the electron density map of the gS87 fibril, the unassigned island was observed on the outer surface of the fibril core, which was adjacent to β2. **b** Cross-section view for

the density map with a built-in structure model of gS87 α-syn, and the measured distances between A89 & island and E46 & K58 (~40 Å for 12 residues). **c** gS87 density maps of different threshold values with extra densities marked. **d** Zoom-in views of hydrophilic zipper-like interactions in gS87 fibril structure to the stabilization of the U-shaped structure. **e** Zoom-in views of the hydrophobic interactions in gS87 fibril model. Residues involved in the interactions are indicated in spheres. **f** The structure of the pS87 fibril, with the salt bridge between K32 and E46, the hydrogen bond between K34 and Y39, and the steric zipper-like hydrophobic interaction shown in the zoom-in views.

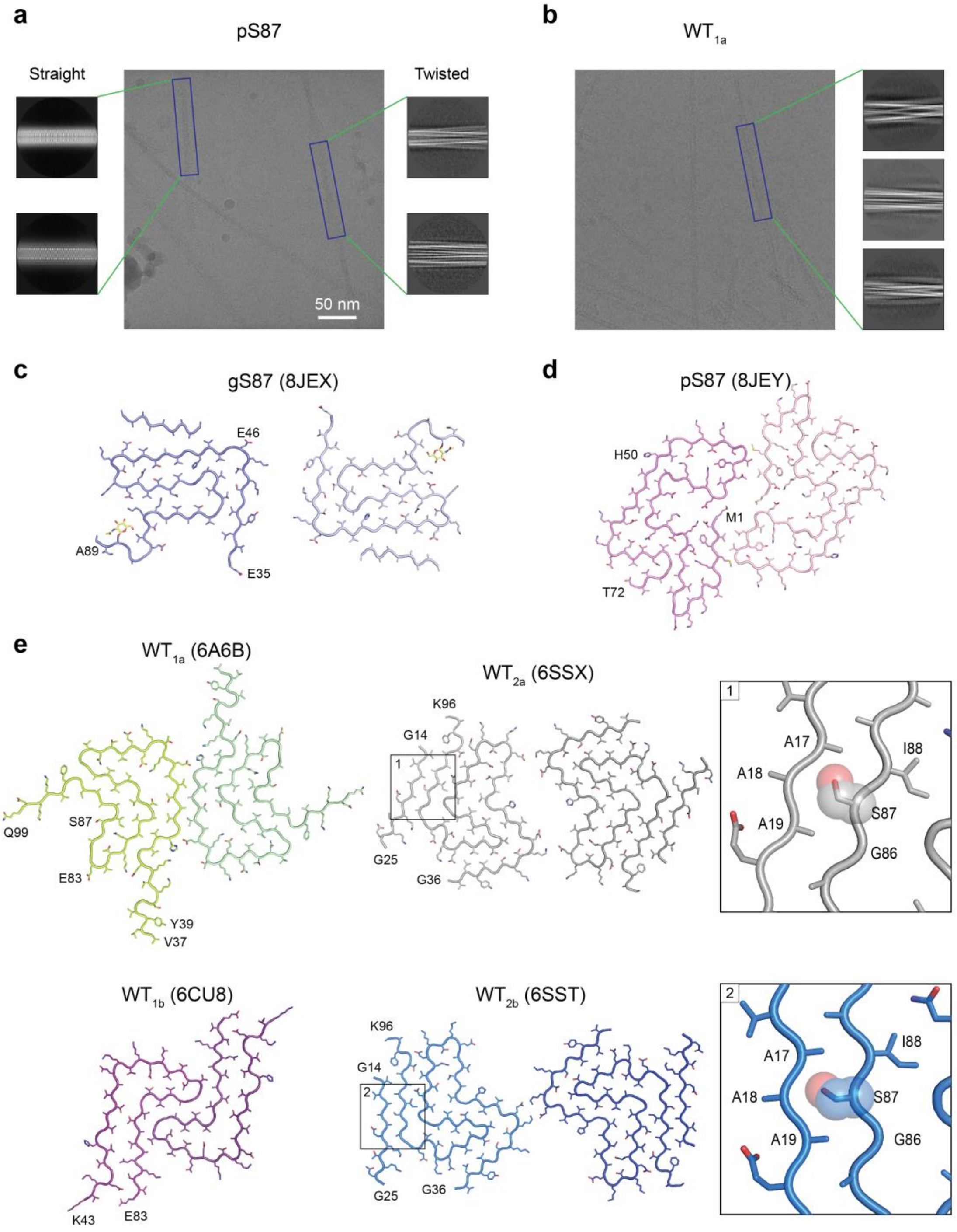

Supplementary Fig. 7. Structural comparison of the gS87, pS87 and different WT fibril polymorphs.

a 2D classification averages of straight polymorph (left) and twisted polymorph (right) of pS87 fibril. **b** 2D classification averages of WT polymorph 1a (WT_{1a}). **c**, **d** The structural model of gS87 fibril (**c**) and pS87 fibril (**d**). **e** The structure of unmodified WT α-syn fibrils (Polymorph 1a, 1b, 2a and 2b) with their PDB codes (top), and the conformation of S87 shown in the zoom-in views (bottom).

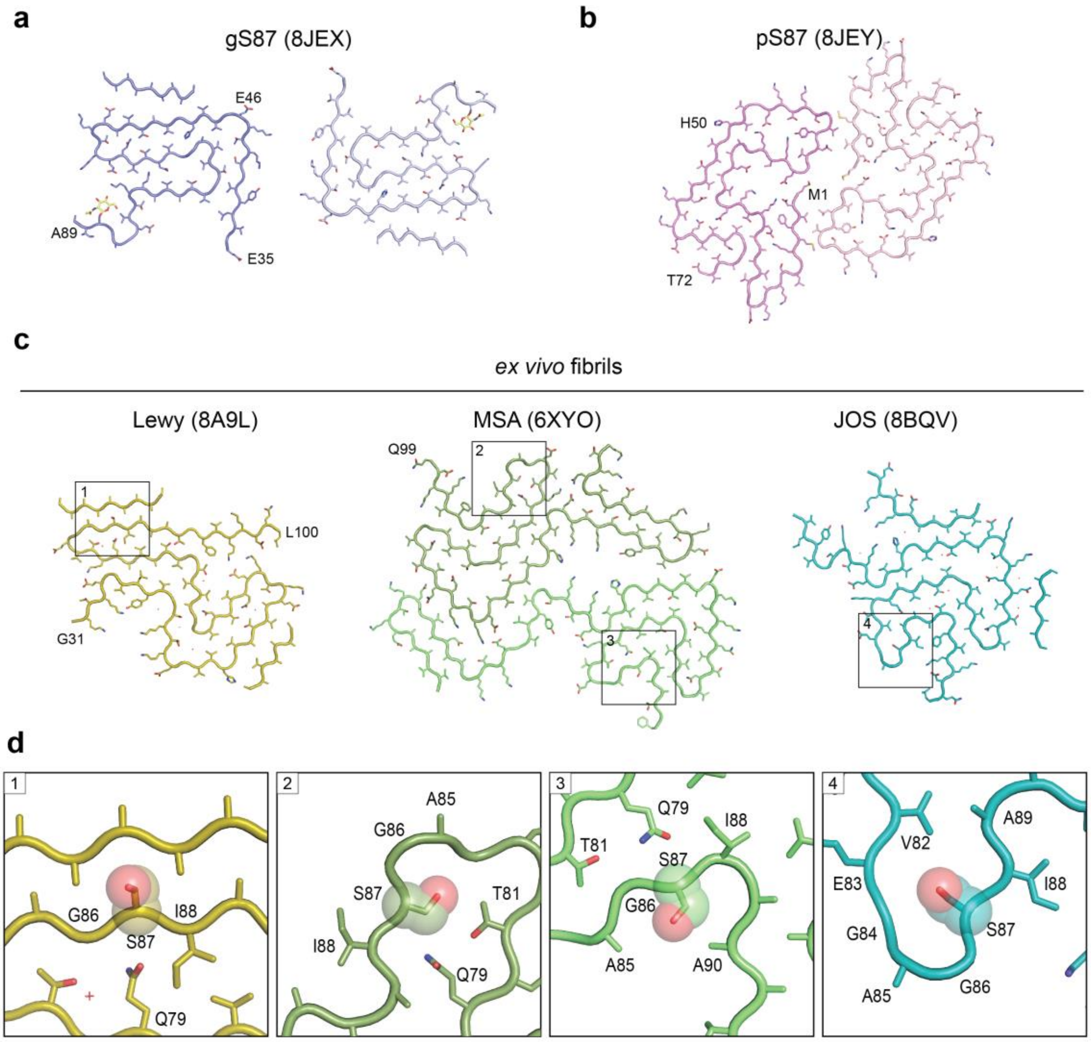

Supplementary Fig. 8. Structural analysis of the gS87, pS87 and *ex vivo* α-syn fibrils.

a, b The structural model of gS87 fibril (**a**) and pS87 fibril (**b**). **c** The structural model of *ex vivo* fibrils: Lewy fold (PDB: 8A9L), MSA fold (PDB: 6XYO) and JOS fold (PDB: 8BQV). **d** Zoom-in views of conformation of S87 in (**c**).

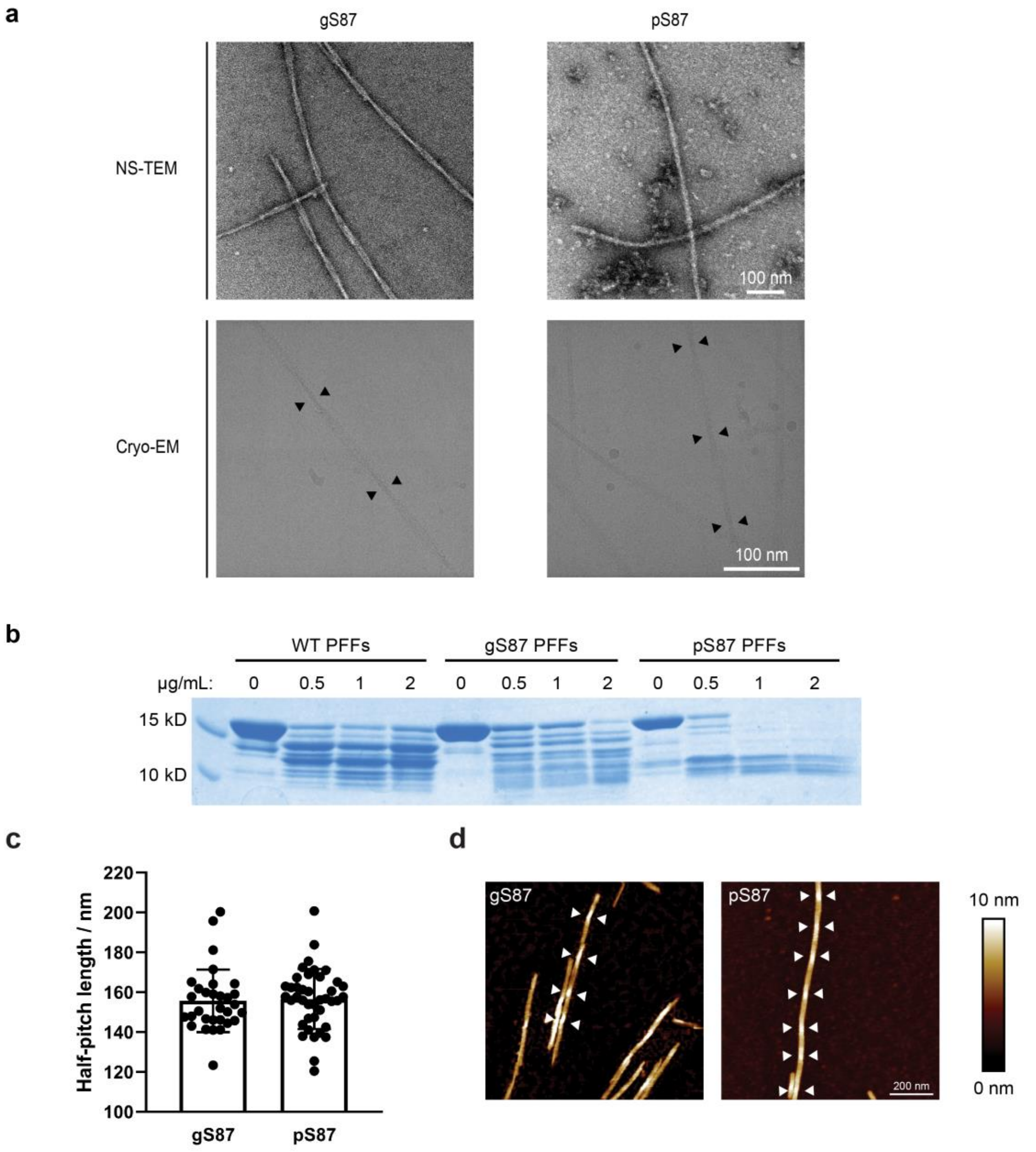

Supplementary Fig. 9. Characterization of unmodified WT, gS87 and pS87 fibrils. **a** The fibrils of gS87 (left panel) and pS87 (right panel) fibrils characterized by NS-TEM (top) and cryo-EM (bottom) with twisted fibrils marked. Scale bar: 100 nm. **b** unmodified WT, gS87 and pS87 PFFs were incubated with proteinase K with different concentrations as indicated at 37°C for 20 min. **c** The AFM statistics of gS87 and pS87 half-pitch length with the mean of 156 nm, 157 nm, respectively. Data correspond to mean ± s.d., n = 31 (gS87), n = 40 (pS87). **d** AFM characterization gS87 and pS87 fibrils with half-pitch marked. Scale bar: 200 nm. Source data are provided as a Source Data file.

Supplementary Table 1. Cryo-EM data collection, modeling and refinement statistics.

Data collection and processing	**gS87 α-syn**<br>(EMD: 36202)<br>(PDB: 8JEX)	**pS87 α-syn**<br>(EMD: 36203)<br>(PDB: 8JEY)
**Data Collection**		
Magnification (×)	105,000	105,000
Pixel size (Å)	0.83	0.83
Defocus Range (μm)	-1.0 to -2.0	-1.0 to -2.0
Voltage (kV)	300	300
Camera	BioContinuum K3	BioContinuum K3
Microscope	Krios G4	Krios G4
Exposure time (s/frame)	0.05	0.05
Number of frames	40	40
Total dose ($e^-/Å^2$)	55	55
**Reconstruction**		
Micrographs	2,134	2,423
Manually picked fibrils	21,328	27,806
Box size (pixel)	360	360
Inter-box distance (Å)	30	30
Initial particle images (no.)	465,930	647,678
Final particle images (no.)	24,910	61,047
Resolution (Å)	3.1	2.6
Map sharpening B-factor ($Å^2$)	-96.50	-86.47
Helical rise (Å)	-179.72	-179.72
Helical twist (°)	2.41	2.41
**Atomic model**		
Non-hydrogen atoms	2,616	3,060
Protein residues	384	432
Ligands	6	0
r.m.s.d. Bond lengths	0.008	0.004
r.m.s.d. Bond angles	0.986	0.746
All-atom clash score	4.96	4.75
Rotamer outliers	0 %	0 %
Ramachandran Outliers	0 %	0 %
Ramachandran Allowed	3.33 %	4.29 %
Ramachandran Favored	96.67 %	95.71 %
