# Supplementary material for: Phosphorylation and O-GlcNAcylation at the same α-synuclein site generate distinct fibril structures
Source: Nat Commun. 2024 Mar 27;15:2677. doi: 10.1038/s41467-024-46898-1 (PMC10973503; doi:10.1038/s41467-024-46898-1)
Supplement: Supplementary file 3 — Reporting Summary [file 41467_2024_46898_MOESM3_ESM.pdf]

nature portfolio

Corresponding author(s): Cong Liu & Yan-Mei Li
Last updated by author(s): Feb 23, 2024

Reporting Summary

Nature Portfolio wishes to improve the reproducibility of the work that we publish. This form provides structure for consistency and transparency in reporting. For further information on Nature Portfolio policies, see our Editorial Policies and the Editorial Policy Checklist.

Statistics

For all statistical analyses, confirm that the following items are present in the figure legend, table legend, main text, or Methods section.

n/a	Confirmed	
☐	☒	The exact sample size (*n*) for each experimental group/condition, given as a discrete number and unit of measurement
☐	☒	A statement on whether measurements were taken from distinct samples or whether the same sample was measured repeatedly
☐	☒	The statistical test(s) used AND whether they are one- or two-sided<br>*Only common tests should be described solely by name; describe more complex techniques in the Methods section.*
☒	☐	A description of all covariates tested
☒	☐	A description of any assumptions or corrections, such as tests of normality and adjustment for multiple comparisons
☐	☒	A full description of the statistical parameters including central tendency (e.g. means) or other basic estimates (e.g. regression coefficient) AND variation (e.g. standard deviation) or associated estimates of uncertainty (e.g. confidence intervals)
☐	☒	For null hypothesis testing, the test statistic (e.g. *F*, *t*, *r*) with confidence intervals, effect sizes, degrees of freedom and *P* value noted<br>*Give P values as exact values whenever suitable.*
☒	☐	For Bayesian analysis, information on the choice of priors and Markov chain Monte Carlo settings
☒	☐	For hierarchical and complex designs, identification of the appropriate level for tests and full reporting of outcomes
☒	☐	Estimates of effect sizes (e.g. Cohen's *d*, Pearson's *r*), indicating how they were calculated

Our web collection on statistics for biologists contains articles on many of the points above.

Software and code

Policy information about availability of computer code

Data collection	Cryo-EM: EPU 2 (Thermo Fisher); HPLC: LabSolutions 5.98 & 5.111; Mass: Chameleon 7.2.10; NMR: delta v4.3.3
Data analysis	Relion v3.1, MotionCor2 v1.2.1, CTFFIND v4.1.8, COOT v0.8.9.2, PHENIX v1.13, UCSF Chimera v1.15 and pymol v2.5, GraphPad Prism 8, graphpad prism 9, NanoScope Analysis v1.5, MestReNova v9.0.1

For manuscripts utilizing custom algorithms or software that are central to the research but not yet described in published literature, software must be made available to editors and reviewers. We strongly encourage code deposition in a community repository (e.g. GitHub). See the Nature Portfolio guidelines for submitting code & software for further information.

Data

Policy information about availability of data

All manuscripts must include a data availability statement. This statement should provide the following information, where applicable:

- Accession codes, unique identifiers, or web links for publicly available datasets
- A description of any restrictions on data availability
- For clinical datasets or third party data, please ensure that the statement adheres to our policy

Density maps of double filament of gS87 α-syn fibril and twisted filament pS87 α-syn fibril are available in Electron Microscopy Data Bank (EMDB) with entry codes: EMD-36202[https://www.ebi.ac.uk/pdbe/entry/emdb/EMD-36202] for double filament of gS87 α-syn fibril and EMD-36203[https://www.ebi.ac.uk/pdbe/entry/emdb/EMD-36203] for twisted filament pS87 α-syn fibril. And the structure models have been deposited in the Protein Data Bank (PDB) with entry codes: 8JEX [http://doi.org/10.2210/pdb8JEX/pdb] for double filament of gS87 α-syn fibril and 8JEY[http://doi.org/10.2210/pdb8JEY/pdb] for twisted filament pS87 α-syn fibril.

The PDB codes for WT1a, WT2b, WT2a, WT2b, pY39, Lewy fold, MSA fold, and JOS fold are 6A6B[http://doi.org/10.2210/pdb6A6B/pdb], 6CU8[http://doi.org/10.2210/pdb6CU8/pdb], 6SSX[http://doi.org/10.2210/pdb6SSX/pdb], 6SST[http://doi.org/10.2210/pdb6SST/pdb], 6L1T[http://doi.org/10.2210/pdb6L1T/pdb], 8A9L[http://doi.org/10.2210/pdb8A9L/pdb], 6XYO[http://doi.org/10.2210/pdb6XYO/pdb], and 8BQV[http://doi.org/10.2210/pdb8BQV/pdb], respectively. All data needed to evaluate the findings of this study are also available from the corresponding author upon reasonable request. Source Data are provided with this paper.

Research involving human participants, their data, or biological material

Policy information about studies with human participants or human data. See also policy information about sex, gender (identity/presentation), and sexual orientation and race, ethnicity and racism.

Reporting on sex and gender	N/A
Reporting on race, ethnicity, or other socially relevant groupings	N/A
Population characteristics	N/A
Recruitment	N/A
Ethics oversight	N/A

Note that full information on the approval of the study protocol must also be provided in the manuscript.

Field-specific reporting

Please select the one below that is the best fit for your research. If you are not sure, read the appropriate sections before making your selection.

☒ Life sciences ☐ Behavioural & social sciences ☐ Ecological, evolutionary & environmental sciences

For a reference copy of the document with all sections, see nature.com/documents/nr-reporting-summary-flat.pdf

Life sciences study design

All studies must disclose on these points even when the disclosure is negative.

Sample size	Experiments described in this study were performed with at least 3-6 samples for each group. In detail, n=3 for ThT kinetic assay in Figure 1b, fibrils formed by gS87, pS87 and WT in >3 independent experiments provide reproducible images and their data were analyzed in Nanoscope software in Figure 1c, n=20 for pS129 α-syn aggregation induced by different types of fibrils in Figure 5c, n=3 independent samples for cytotoxicity measurement in Figure 5d, and n=31(gS87), n=40(pS87) for half-pitch length in Supplementary 9c.
Data exclusions	None.
Replication	At least three independent biological repeats were performed. All attempts at replication were successful.
Randomization	Randomization is not applicable for the experiments in this structural study. Purified protein and semi-synthetic protein were used in this study and no animal or human studies were involved.
Blinding	Since there were no animal or human study involved in the study, blinding is not applicable for the experiments in this structural study.

Reporting for specific materials, systems and methods

We require information from authors about some types of materials, experimental systems and methods used in many studies. Here, indicate whether each material, system or method listed is relevant to your study. If you are not sure if a list item applies to your research, read the appropriate section before selecting a response.

Materials & experimental systems

n/a	Involved in the study	
☐	☒	Antibodies
☒	☐	Eukaryotic cell lines
☒	☐	Palaeontology and archaeology
☐	☒	Animals and other organisms
☒	☐	Clinical data
☒	☐	Dual use research of concern
☒	☐	Plants

Methods

n/a	Involved in the study	
☒	☐	ChIP-seq
☒	☐	Flow cytometry
☒	☐	MRI-based neuroimaging

Antibodies

Antibodies used	anti-phospho-α-synuclein (S129) (1:1,000, Abcam, Cat.# ab51253); anti-MAP2 (1:2500, Abcam, Cat.# ab5392); antibodies of Alexa Fluor 488- and Alexa Fluor 568- (1:1000, Invitrogen, 2420700, 2155282, respectively)
Validation	The antibodies are well validated for the indicated use by the manufacturer available on their websites.

Animals and other research organisms

Policy information about studies involving animals; ARRIVE guidelines recommended for reporting animal research, and Sex and Gender in Research

Laboratory animals	Embryonic day 16–18 Sprague-Dawley rats embryos used in this paper were purchased from Shanghai SIPPR BK Laboratory Animals Ltd, China.
Wild animals	None.
Reporting on sex	Sex is not considered in the design.
Field-collected samples	None.
Ethics oversight	All animal and cell experiments in this study were performed following the protocols approved by the Animal Care Committee of the Interdisciplinary Research Center on Biology and Chemistry (IRCBC), Chinese Academy of Sciences (CAS). Embryonic day 16–18 Sprague-Dawley rats embryos used in this paper were purchased from Shanghai SIPPR BK Laboratory Animals Ltd, China.

Note that full information on the approval of the study protocol must also be provided in the manuscript.
